# Automated detection of Kaposi sarcoma-associated herpesvirus infected cells in immunohistochemical images of skin biopsies

**DOI:** 10.21203/rs.3.rs-4736178/v1

**Published:** 2024-08-17

**Authors:** Iftak Hussain, Juan Boza, Robert Lukande, Racheal Ayanga, Aggrey Semeere, Ethel Cesarman, Jeffrey Martin, Toby Maurer, David Erickson

## Abstract

Immunohistochemical (IHC) staining for the antigen of Kaposi sarcoma-associated herpesvirus (KSHV), latency-associated nuclear antigen (LANA), is helpful in diagnosing Kaposi sarcoma (KS). A challenge, however, lies in distinguishing anti-LANA-positive cells from morphologically similar brown counterparts. In this work, we demonstrate a framework for automated localization and quantification of LANA positivity in whole slide images (WSI) of skin biopsies, leveraging weakly supervised multiple instance learning (MIL) while reducing false positive predictions by introducing a novel morphology-based slide aggregation method. Our framework generates interpretable heatmaps, offering insights into precise anti-LANA-positive cell localization within WSIs and a quantitative value for the percentage of positive tiles, which may assist with histological subtyping. We trained and tested our framework with an anti-LANA-stained KS pathology dataset prepared by pathologists in the United States from skin biopsies of KS-suspected patients investigated in Uganda. We achieved an area under the receiver operating characteristic curve (AUC) of 0.99 with a sensitivity and specificity of 98.15% and 96.00% in predicting anti-LANA-positive WSIs in a test dataset. We believe that the framework can provide promise for automated detection of LANA in skin biopsies, which may be especially impactful in resource-limited areas that lack trained pathologists.

### Introduction

KS refers to a form of cancer most commonly affecting the skin or mucous membrane, caused by the Kaposi sarcoma-associated herpesvirus (KSHV; also known as human herpesvirus 8 (HHV-8) [1]. Its development is frequently driven by immune suppression from HIV infection[2]. If a clinician suspects KS, then a biopsy and histopathologic evaluation is the next step in diagnosis. Although conventional hematoxylin and eosin (H&E) staining alone can be used to confirm or refute the diagnosis of KS, IHC stain for a specific protein derived from KSHV, known as latency-associated nuclear antigen (LANA), can be helpful in assisting pathologic interpretation[3].

IHC for LANA is typically achieved using colored labels such as brown chromogen. This color, however, can be confused with other brown counterparts, such as cytoplasmic haemosiderin or melanin[3]. These are common, given that a feature of KS is red blood cell extravasation that leads to hemoglobin release and haemosiderin accumulation in histiocytes, and the presence of melanin in skin, where KS is frequently found. These can be distinguished from LANA positivity by IHC, given their cytoplasmic localization and appearance of the pigments, while LANA should be considered positive only when a distinct punctate nuclear pattern is observed in the cell. However, an experienced surgical pathologist or dermatopathologist is most likely to make this distinction, which requires meticulous microscopic observation over the pathology slide. In addition, this method solely depends on the observer's visual perception, which may vary from one pathologist to another based on their training and experience[4–6]. There may be a lack of pathology experience with KS in settings where it is not very common, or there may be a complete insufficiency of pathologists where this disease is most common, particularly in Sub-

Equatorial Africa. Therefore, an automated framework that accurately detects and quantifies anti-LANA-positivity from pathology slides can facilitate accurate, high-throughput, and timely diagnosis.

Recent years have witnessed a growing interest in applying artificial intelligence (AI) techniques to WSIs for various tasks, including prognosis prediction, cancer classification, and genetic status prediction[7–12]. Specifically, convolutional neural networks (CNN), a form of deep learning, have been extensively used to automate tasks in histopathology at a level comparable to that of experienced pathologists. These studies demonstrated the efficacy of computational pathology in clinical decision support[13,14]. DL-based strategies were proven successful in recognizing particular cell types and cancer markers in IHC-stained WSIs[15–17]. In most of these studies, pathologist-level interpretations were obtained through supervised learning, which required pixel-level annotations prepared by expert pathologists. Considering the large size of WSIs, particularly in the case of KS, localizing and annotating individual anti-LANA-positive cells is highly time-consuming and costly. To overcome these limitations, weakly supervised learning can reduce the annotation requirement by automatically inferring pixel-level information from slide-level annotations. Multiple instance learning (MIL), a method frequently used within weakly supervised learning framework, was recently demonstrated for localizing tumors in pathology slides prepared from biopsies of prostate cancer, basal cell carcinoma, and breast cancer metastases to axillary lymph nodes, which resulted in areas under the curve above 0.98 for all cancer types[18,19]. It should be feasible to develop a weakly-supervised deep learning framework based on MIL for localization and classification of anti-LANA-positive cells in a WSI. Considering the MIL approach, if all the tiles of the WSI are negative for anti-LANA staining, then the pathology slide is negative. In contrast, if a single tile is positive for anti-LANA staining, the pathology slide is positive.

Herein, we demonstrate an automated framework by leveraging a MIL-based weakly-supervised training approach to train a network capable of localizing and quantifying LANA positivity in whole slide images. The developed framework produces interpretable heatmaps indicating the most probable tiles within the WSI where an anti-LANA-positive cell is likely to be found. Since, in the MIL-based approach, a single spurious classification results in a false positive, the inclusion of a slide aggregation method on top of the MIL is necessary. We implemented a novel slide aggregation method by learning distinguishable morphological features at the tile level through a random forest machine-learning classifier. Overall, the developed framework provides a platform for the fast and accurate detection of LANA positivity in IHC-stained skin biopsy specimens.

### Methods

## Dataset

This study is IRB-approved by Makerere University (Study Number: SBS-495), The School of Biomedical Sciences Research Ethics Committee. All methods were performed in accordance with the relevant guidelines and regulations of the IRB. The consent was informed, and all of the participants signed written informed consent forms before collecting the biopsy samples. The skin tissue biopsy samples were collected over a period of 14 months (January 25, 2017 – April 03, 2018) from patients in Uganda suspected of having KS. The biopsy material was embedded into paraffin blocks in Uganda, and sections for anti-LANA IHC staining were prepared at Weill Cornell Medicine using an automated IHC histology stainer (Leica Bond III system). The resulting slides were scanned with a digital pathology slide scanner (Aperio GT450) and were saved in a pyramidal format at various magnifications. An initial blinded pathologic assessment for LANA positivity was conducted at Weill Cornell Medicine, followed by a secondary review by a dermatopathologist at the University of California, San Francisco. For each slide, the de-identified slide number, the percentage of anti-LANA positive cells determined by the pathologists, and the interpretation for LANA positivity (LANA positive or negative) were recorded. This is a retrospective study, all scanned WSIs were anonymized and were accessed from an internal server of Weill Cornell Medicine on May 25, 2023. We do not have access to information that could identify individual participants during or after data collection.

We prepared two independent WSI datasets: the first (Dataset 1) containing 264 WSIs from the 2017 cohort and the second (Dataset 2) with 80 WSIs from the 2018 cohort. Dataset 1, with 158 anti-LANA-positive and 106 anti-LANA-negative cases, was split evenly for training and validation. The validation set was further divided into two equal subsets: one for initial model assessments (Validation Dataset A) and another for final assessments (Validation Dataset B). Dataset 2 served as the testing set. Each WSI filename was suffixed with '1' for anti-LANA-positive and '0' for anti-LANA-negative to facilitate automatic slide labeling during the training and validation phases.

## MIL approach for model training

As illustrated in Fig. 1a, we treated each slide as an independent "bag" with a binary label (0 or 1), representing its classification as either anti-LANA-negative or positive. Within each bag, instances were defined as tiles measuring 224 x 224 pixels, extracted at 40X magnification to capture cellular-level morphology. A VGG19 architecture pre-trained its weights on the ImageNet dataset was used for both inference and training. The final fully connected layer is customized to suit the binary classification task, with two output features specifically designed to classify instances into positive and negative classes. During inference, each batch of tiles was passed through the model sequentially to obtain logits, representing raw scores for each class. These logits are then converted into probabilities using a SoftMax activation function. After obtaining probabilities, tiles within slides are ranked based on their probability scores, reflecting their likelihood of contributing to the classification of the slide. For slides labeled as anti-LANA-positive, tiles are ranked primarily based on their probability of belonging to the positive class, with higher scores indicating a more substantial likelihood of positively influencing the slide's classification. Conversely, for slides labeled as anti-LANA-negative, tiles are ranked based on their probability scores for the negative class, reflecting their potential to reinforce the slide's negative classification. After ranking, top-k tiles from each slide were used to re-train the model using the Adam optimizer, which dynamically adjusted the learning rates for each parameter during training. This optimization process aimed to update the model's weights to maximize the probability of instances selected from anti-LANA-positive slides while minimizing the probability of instances selected from anti-

LANA-negative slides. Model checkpoints were implemented to save the best weights during training for 100 epochs. We found that using a learning rate of 1e-4, batch size of 512, and training for 25 epochs were enough to obtain a model to classify WSIs with low loss and high accuracy.

The prediction through the MIL model was achieved by processing all tiles associated with each slide during validation or testing depicted with the workflow shown in Fig. 1b. Utilizing a probability threshold value of 0.5, the model determines the slide label based on the presence of anti-LANA-positive tiles within the slide. If at least one tile is classified as positive, the entire slide is classified as positive; conversely, if all tiles are classified as negative, the slide is anti-LANA-negative. To estimate the probability of a slide being positive, a max-pooling technique over the tile probabilities is employed, selecting the highest probability among all tiles within the slide as the probability of being positive. A major disadvantage of the MIL framework is that it lacks robustness due to the max-pooling operation utilized in slide prediction. A single erroneous classification can significantly alter the slide prediction outcome, potentially leading to an increased number of false positives[19]. It is evident from the confusion matrix yielded by the MIL framework in predicting WSIs in the validation dataset A, indicating a sensitivity of 98% and specificity of 88% in predicting WSIs (Fig. 2a). This is attributed to the existence of brown-colored substances mimicking anti-LANA-positive cells in dermatopathology cases other than KS as shown in Fig. 2b. Usually, a slide aggregation model is developed on top of the MIL classification by developing machine-learning classifiers based on extracted features from the probability heatmap or number of tiles per class to mitigate this type of problem[20].

We developed a novel slide aggregation model leveraging the distinctive morphological and proliferative attributes associated with anti-LANA expression in LANA-positive WSIs. By utilizing curated evaluation reports from pathologists, we constructed a dataset for feature extraction. This dataset includes WSIs identified as anti-LANA-positive, with a minimum of 20% positive cells, and all the anti-LANA-negative WSIs previously predicted as positive by the MIL model in validation dataset A. After predictions were made on the WSIs in validation dataset A by the MIL model, we identified the top 15 individual tiles predicted as anti-LANA-positive along with their coordinates, as illustrated in Fig. 3. We recorded the MIL prediction probabilities of individual tiles as a feature. To mitigate false positive predictions, primarily caused by brown-colored substances present in a tile, we employed color deconvolution using a well-established algorithm to extract the IHC channel and the hematoxylin (H) channel. The mean pixel intensity of the IHC and H channels served as features for individual tiles, along with their standard deviation (SD) calculated for the entire tile group, which was used as a WSI feature. Additionally, we extracted the red (R), green (G), and blue (B) channels from individual tiles to utilize their mean intensity as a feature. Their SD for the overall group was employed as a representative WSI feature. Utilizing the tile coordinates, we computed the distances of individual positive tiles from the other 14 tiles within the WSI and stored them as individual arrays. These arrays facilitated the evaluation of the minimum distance to a neighboring positive tile from each individual tile, with the minimum distance being used as a representative feature. Finally, the SD of the minimum distances for the entire anti-LANA-positive tile group was utilized as a representative WSI feature.

We used these 13 extracted features to train four broadly used machine-learning classifiers: random Forest, K-nearest neighbors, naive bayes, and support vector machine. We used the validation dataset B to optimize our trained machine-learning models. The accuracy of the Random Forest classifier was found to be the highest, with an area under the ROC curve (AUC) of 0.985 (Fig. 4a). We used the trained random forest classifier as the slide aggregation model for the rest of the analysis. It is evident from the feature importance plot (Fig. 4b) for the random forest classifier that the spatial localization and mean IHC channel intensity of the tile in the WSI contribute primarily to differentiating true positives from false positives.

Figure 1c illustrates the final developed framework. After obtaining initial predictions from the MIL model, the framework automatically extracts representative features from the predicted anti-LANA-positive tiles, which undergo classification through the random forest classifier. Similar to the MIL model, we used the maximum probability of the predicted tiles as a representative for the WSI. We used a probability threshold 0.5 to classify the WSI as anti-LANA-positive or negative. The framework provides the % of anti-LANA-positive tiles, which is calculated using the following equation:

$$\text{\% of anti-LANA-positive tiles} = \frac{L_+}{L_+ + L_-} \times 100\% \qquad (1)$$

where $L_+$ is the number of predicted positive tiles and $L_-$ is the number of predicted negative tiles by the framework. We also integrated gradient weighted class activation map or Grad-CAM to visualize the model prediction confidence on the anti-LANA-positive tiles, which were later superimposed for each positive tile to generate the WSI-heatmap indicating areas where our framework is confident of finding anti-LANA-positive cells.

## Results

We assessed the performance of our framework using the test dataset, consisting of WSIs from the 2018 cohort, and compared the results with the pathologist's evaluations from the assessment reports. Figure 5a and 5b display the confusion matrix, highlighting the enhancement in identifying false positive cases achieved by transitioning from the initial MIL framework to our customized RF-MIL framework. Our RF-MIL framework demonstrates an impressive 12.5% decrease in false positive cases overall. This improvement is further underscored by the ROC curve depicted in Fig. 5c, illustrating a notable enhancement in the Area Under the Curve (AUC) metric from 0.79 (95% confidence interval of 0.68 to 0.91) to 0.99 (95% confidence interval of 0.97 to 1.00) with the implementation of the RF-MIL framework. The integration of the random forest classifier leveraging the handcrafted tile-level features into the traditional MIL framework is instrumental in mitigating false positives, a common challenge in weakly supervised learning approaches of this nature. A minor fraction of false negatives was identified as an intrinsic characteristic of both frameworks, originating from WSIs captured with insufficient focus and poor staining in the test dataset.

Figure 6 presents a series of heatmaps generated by the RF-MIL framework, showcasing diverse stages of KS within the predicted WSIs. These heatmaps meticulously delineate the spatial distribution of potential anti-LANA-positive cells across the WSIs, each highlighted in zoomed-in regions for clarity. Notably, the heatmaps unveil a nuanced distribution of KSHV-infected cells, manifesting as distinct patterns across different stages of KS. In the instance of tumor stage KS, the heatmaps depict pronounced clustering of KHSV-infected cells, suggestive of localized concentrations within these lesions. Conversely, in plaque and patch stage KS, the distribution appears more dispersed, with KHSV-infected cells sparsely scattered throughout the tissue. Augmenting these spatial insights, the accompanying bar diagram illustrates the percentage of anti-LANA-positive tiles within predicted WSIs. This supplementary visualization offers a quantitative perspective, elucidating the varying degrees of KHSV infection across predicted regions. Together, these analyses provide comprehensive insights into the heterogeneous distribution patterns of KHSV-infected cells across distinct stages of KS, facilitated by the RF-MIL framework's robust computational methodology.

### Discussion

We introduced an innovative MIL-based, weakly supervised deep learning framework for automated localization and quantification of LANA expression in IHC-stained pathology slides. Our framework notably reduces false positives, a common challenge in the MIL approach for weakly supervised learning. By incorporating a random forest classifier leveraging various tile-level morphological features, we have improved accuracy and reduced false-positive outcomes. The use of a unique dataset ensures a comprehensive evaluation and the potential for generalization. Although immunohistochemistry is not widely used in resource-limited settings due to various constraints, including reliance on anti-LANA staining, we must recognize the evolving diagnostic technologies and anticipate future advancements in IHC techniques. Hence, our framework has significant potential for widespread adoption. However, the possibility of false negatives due to technical issues during IHC slide preparation, such as variations in staining intensity and tissue processing, underscores the importance of technical precision in IHC procedures. Addressing these challenges will enhance the framework's accuracy and reliability. Exploring solutions, including the use of generative AI for virtual staining, presents a promising avenue to overcome limitations in resource-limited settings and to reduce false negatives.

In summary, we presented a MIL-based weakly supervised deep learning framework fused with morphological evaluation to automate the localization and quantification of anti-LANA expression in immunochemistry-stained pathology slides. The study acknowledges the false positive prediction through MIL, primarily attributed to brown-colored substances mimicking anti-LANA-positive cells in dermatopathology cases other than KS. To address this, we proposed the integration of a random forest machine-learning classifier as a slide aggregator, demonstrating a reduction in false-positive results and yielding an AUC of 0.99 with a sensitivity and specificity of 98.15% and 96.00%. Despite the current limitations associated with the reliance on immunohistochemistry, which may pose challenges in resource-limited settings, the study anticipates future advancements in diagnostic technologies, including the increased accessibility and feasibility of IHC techniques through the integration of various other AI-powered modalities.

### Declarations

## Competing interests

The authors declare no competing interests.

## Author Contribution

Project conception and design were by I.H., J.B., D.E., and E.C. Data collection and processing were performed by R.L., R.A., A.S., J.M., and T.M. The software programming, statistical analysis, and interpretation were performed by I.H. The manuscript was written by I.H. and all authors reviewed the manuscript.

## Acknowledgement

This work was supported by the National Institute of Health (U01 CA269199, U54 CA254571 and P30AI027763).

## Data Availability

The datasets generated during and/or analyzed during the current study are available from the corresponding author upon reasonable request. Source codes and scripts are available on GitHub (https://github.com/Iftak/AI_LANA_IHC.git).

## Figures

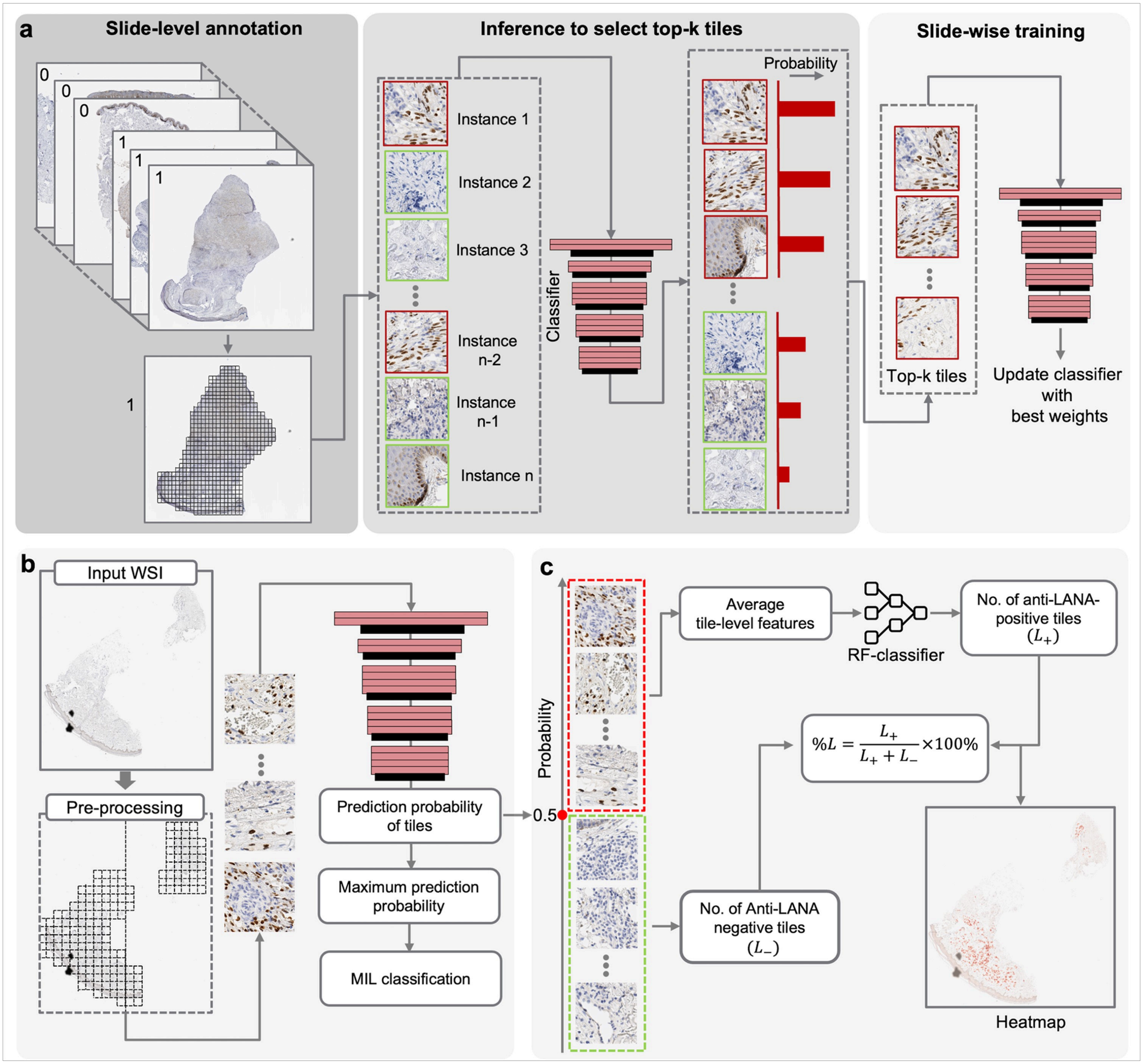

### Figure 1

**Framework for classification of anti-LANA-positive WSIs. a.** An Illustration depicting the treatment of each slide as an independent "bag" for MIL-based training, complete inference pass through the dataset, ranking tiles based on their probability of being positive, and subsequent learning on the top-ranking tiles per slide. **b.**Traditional MIL-based prediction of WSI via max-pooling of tile probabilities. c. Slide aggregation method with the random forest classifier, estimation of % of anti-LANA-positive tiles, and heatmap generation.

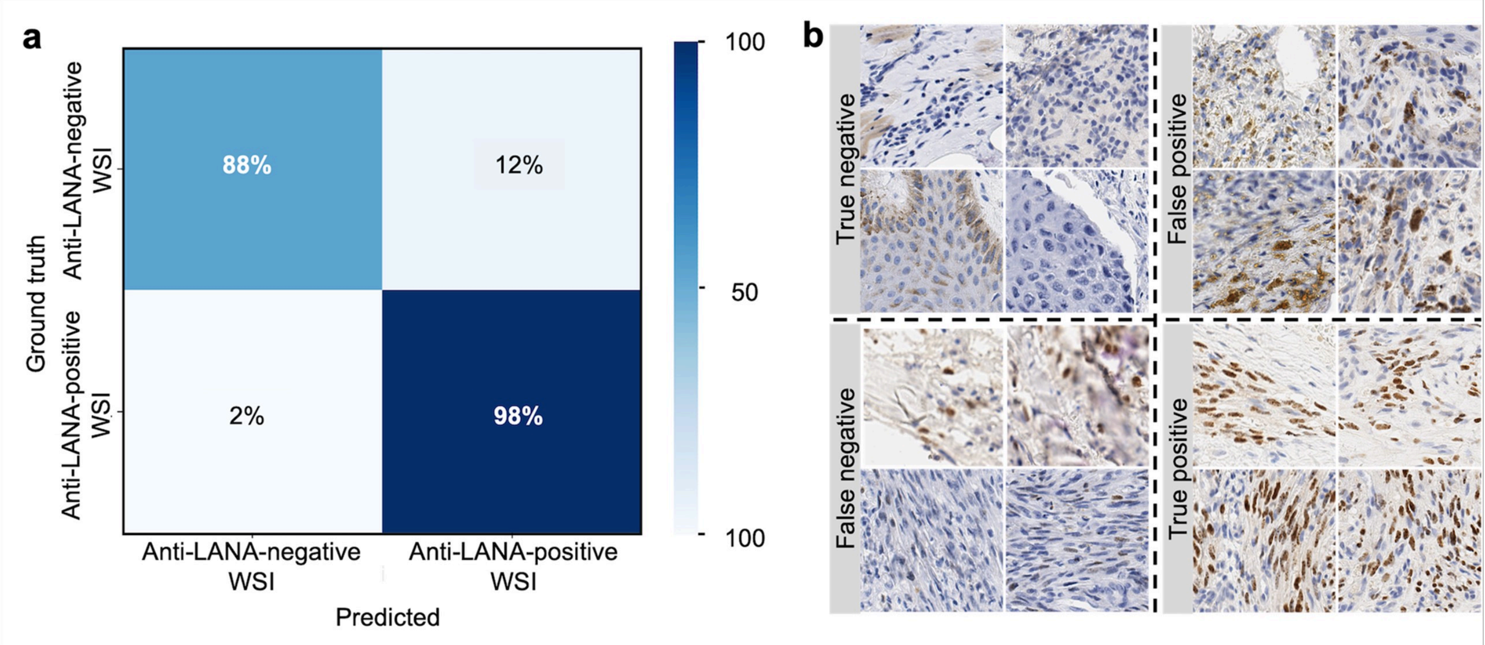

### Figure 2

**Evaluation of MIL Framework with the validation dataset. a.** Confusion matrix depicting the MIL framework's performance in predicting WSIs in validation dataset A. b. Representative tiles from predicted WSIs indicate the generation of false positives due to the presence of brown substances.

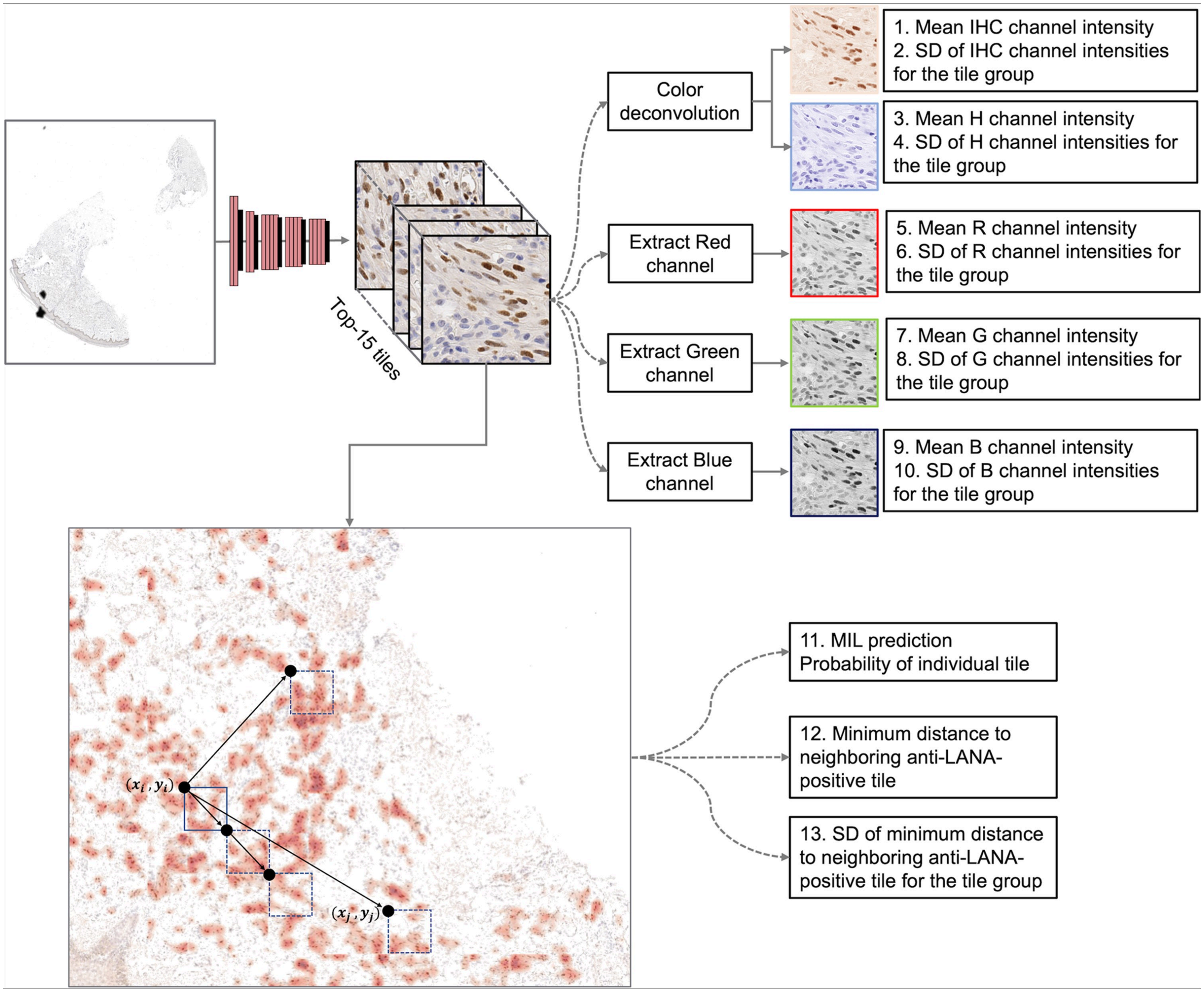

### Figure 3

#### Feature extraction to develop the slide aggregation model.

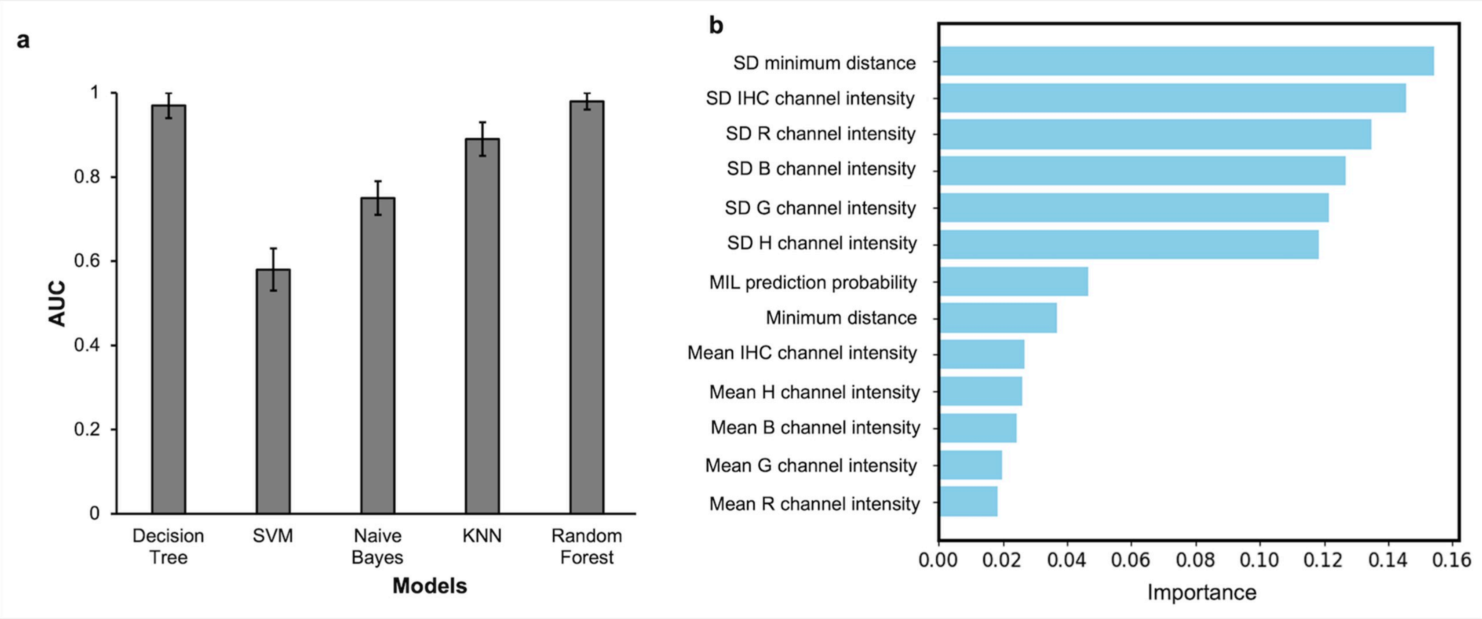

### Figure 4

**Machine-learning classifier. a.** Area under the ROC curve (AUC) of classifying anti-LANA-positive WSIs by different machine-learning models. **b.** Feature importance plot highlights spatial localization and standard deviation of IHC channel intensity as key discriminative factors.

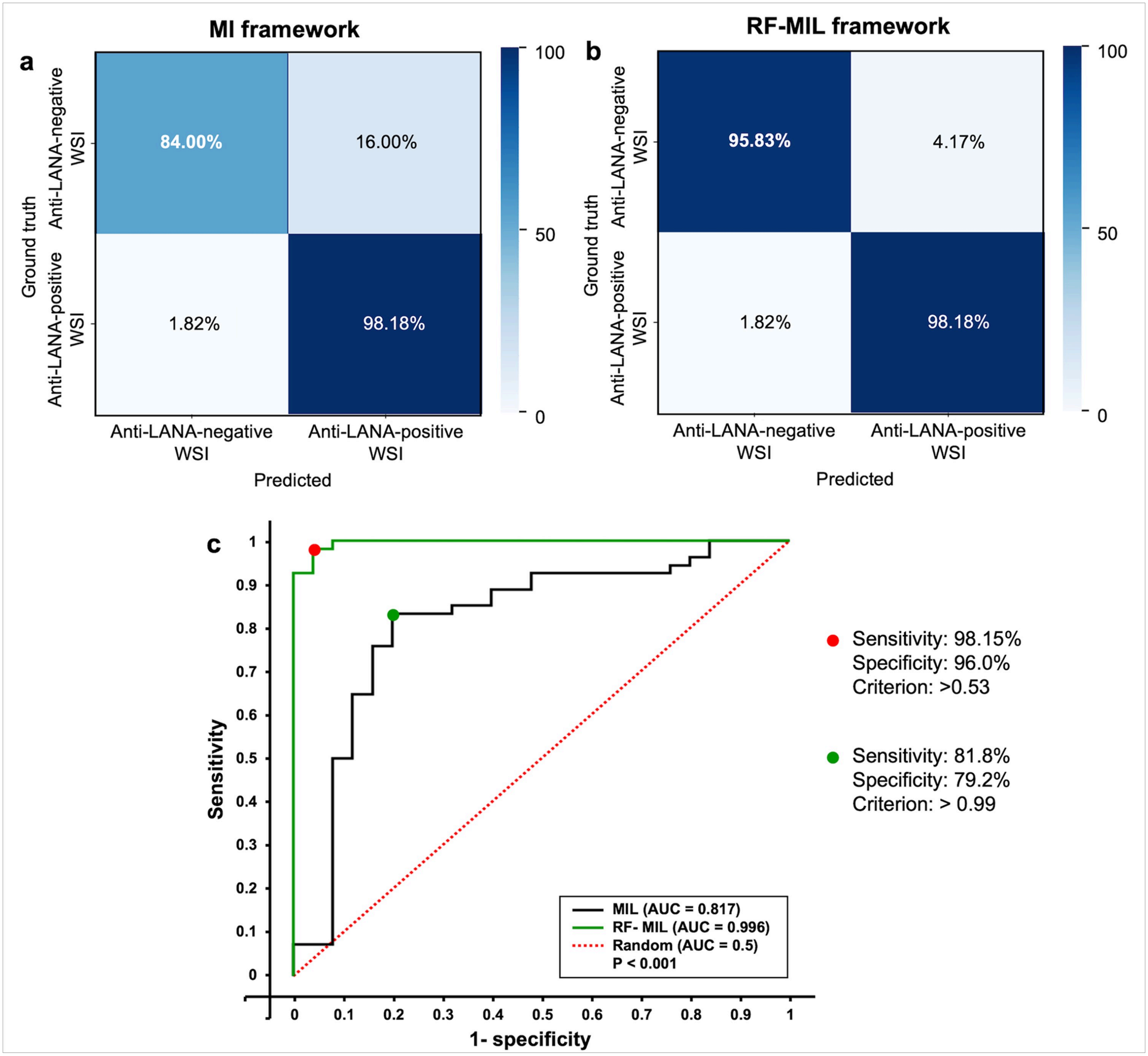

### Figure 5

**Performance Evaluation of RF-MIL Framework on test dataset. a.** and **b.** Confusion matrices generated by MIL and RF-MIL framework for test dataset. **c.** Receiver operating characteristics of the framework in making predictions for anti-LANA-positive WSIs in the test dataset.

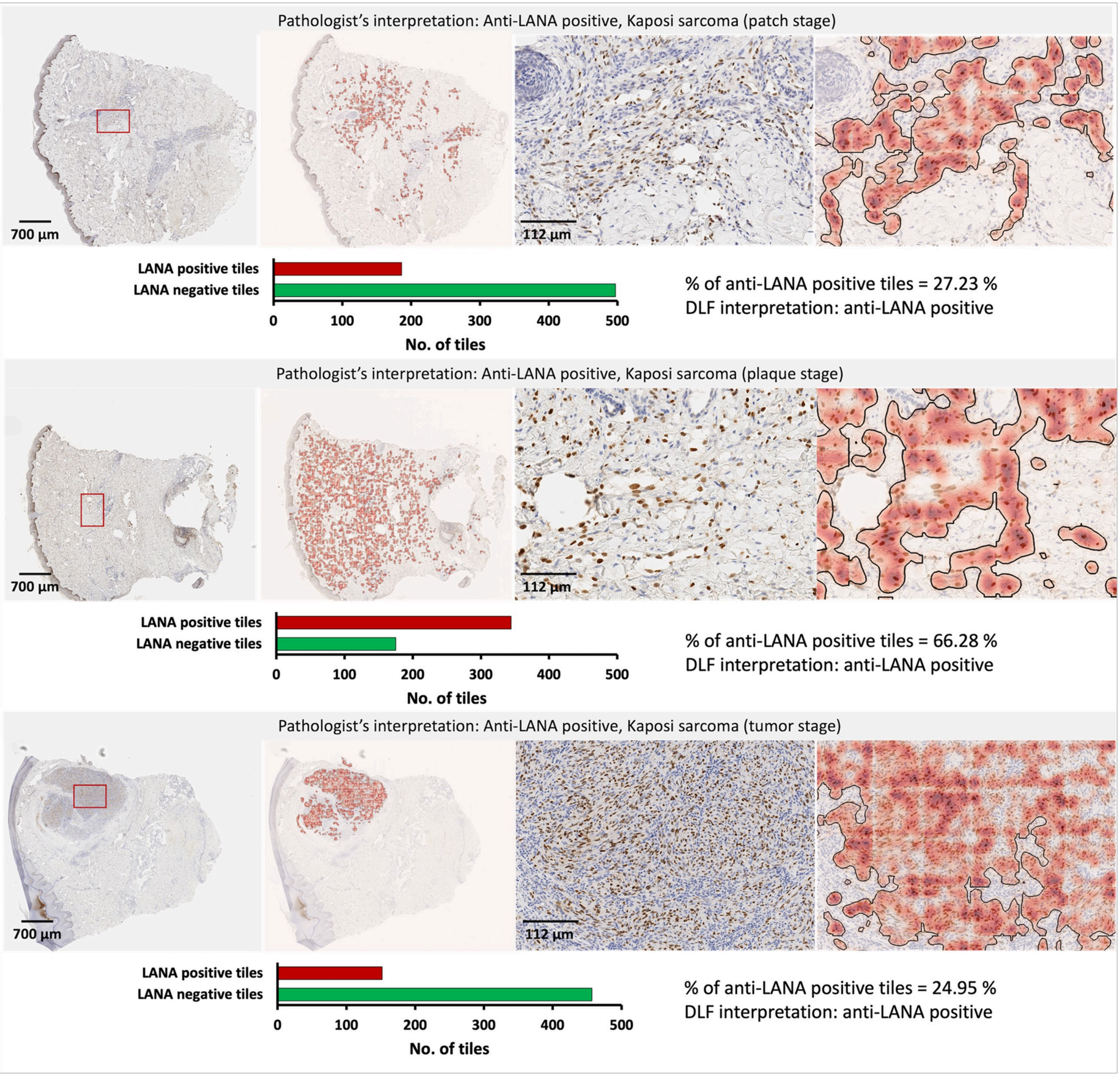

### Figure 6

**Heatmaps generated from the RF-MIL framework.** The heatmaps indicate spatial distribution of anti-LANA-positive cells in diverse Kaposi's Sarcoma stages. The bar diagram quantifies the percentage of positive tiles, offering insights into KHSV infection patterns.
